# Main Group Catalyzed Arene Borylation: Challenges and Opportunities

**DOI:** 10.1021/acscatal.3c01668

**Published:** 2023-05-23

**Authors:** Michael J. Ingleson

**Affiliations:** EaStCHEM School of Chemistry, University of Edinburgh, Edinburgh EH9 3FJ, United Kingdom

C–H borylation is an efficient method to generate organoboranes that are powerful intermediates in synthesis, due in large part to the Suzuki–Miyaura reaction.^1^ Tremendous advances have been made using transition metal based catalysts for arene C–H borylation.^2^ These catalysts generally utilize the variable oxidation states available to the transition metals to effect C–H cleavage and C–B bond formation. Iridium based C–H borylation catalysts in particular are established as one of the foremost methods in the field of C–H functionalization.^3^ Main group compounds also have been applied as catalysts for arene C–H borylation, with the greater abundance, lower cost, and higher permitted exposure limits of most main group elements relative to precious metals one key driver for this research.^4^ A second driver stems from the fact that main group catalysis generally proceeds at a fixed oxidation state thus requiring distinct borylation mechanisms. This can lead to complementary regioselectivity and functional group tolerance to that using transition metal based catalysts. However, significant challenges remain in main group catalyzed arene borylation, particularly around expanding the substrate scope and generating operationally simple methodologies.^5^ Most systems reported to date use (pre)catalysts that are challenging to handle (due to their sensitivity to protic species such as H_2_O) and that are limited in substrate scope to nucleophilic (hetero)arenes (defined herein as substrates with Mayr nucleophilicity values (*N*) > 1, for reference, 2-Me-thiophene and furan have *N* ≈ 1.3).^6^ Identifying a single catalytic system that addresses both of these challenges is essential for the wider uptake of main group catalyzed arene C–H borylation.

This viewpoint summarizes the general approaches used to date in main group catalyzed intermolecular arene C–H borylation. It is not a comprehensive survey of the area; instead, it focuses on analyzing common key steps and through this seeks to highlight some of the current challenges and future opportunities in the field. For stoichiometric (in strong Lewis acid)^7^ and intramolecular C–H borylation, the reader is directed to recent reviews.^8^

## The Importance of [H]^+^

**Scheme 1:**
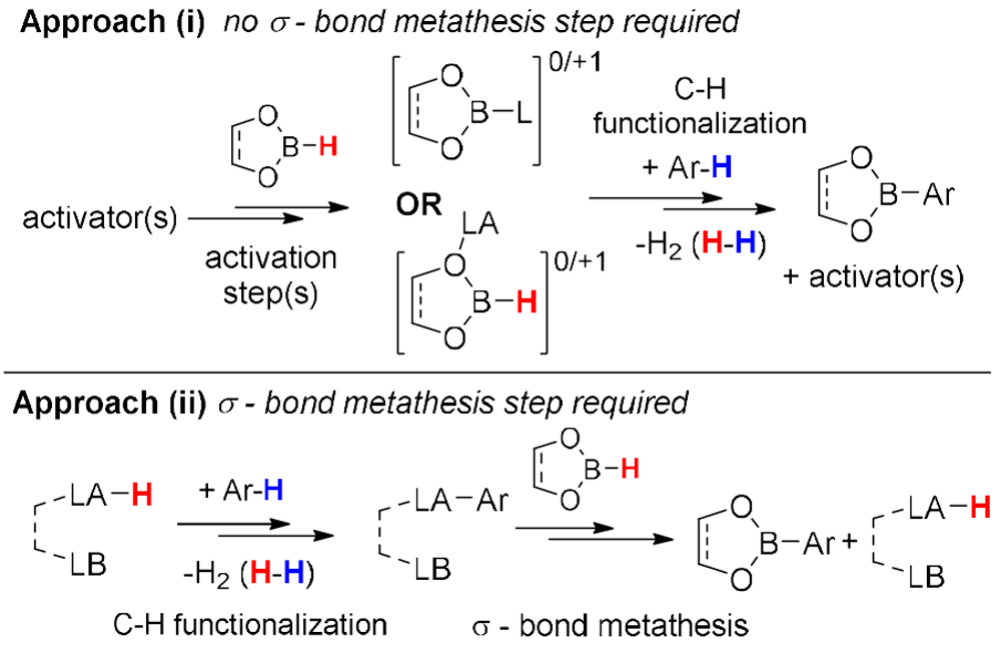
Generic Examples Highlighting the Division of Catalytic Electrophilic Arene C–H Borylation Processes into Two Approaches LA = Lewis acid, LB = Lewis/Brønsted base.

The majority of main group catalyzed arene borylation processes reported to date proceed via a stepwise or a concerted S_E_Ar type process^9^ and thus can be termed electrophilic borylation reactions. In these, controlling the fate of the byproduct from S_E_Ar, [H]^+^, is vital.^10^ Indeed, the effective sequestering of [H]^+^ was recognized as crucial even in the early stoichiometric electrophilic borylation studies from the 1960s.^11^ The generation of [H]^+^ as a byproduct also provides a route to using substoichiometric electrophilic activators in arene borylation. Combining [H]^+^ with a hydroborane (e.g., catecholborane (CatBH) or pinacolborane (PinBH) derivatives) or another hydridic species can lead to H_2_ evolution and (re)generate a reactive electrophile for performing further C–H functionalization. Virtually all main group catalyzed borylation reactions reported to date rely on this as a key step to close the catalytic cycle. To facilitate analysis, these reactions have been divided into two types based on the nature of the electrophile that effects C–H functionalization. Specifically, it separates systems based on whether or not a σ-bond metathesis step is required to form the target aryl-borane post C–H functionalization. These two classes are discussed separately as they have distinct challenges. For example, approach (i) requires converting a hydroborane into an electrophile that effects S_E_Ar (Scheme 1, top), while (ii) uses a separate electrophile to achieve C–H functionalization but then requires a σ-bond metathesis step to close the catalytic cycle (Scheme 1, bottom). Both approaches build upon modern developments in main group chemistry, such as frustrated Lewis pairs (FLPs),^12^ and commonalities are present between them which will be highlighted where appropriate.

## Approach (i) Arene C–H Functionalization by Electrophiles Derived from Hydroboranes

**Scheme 2:**
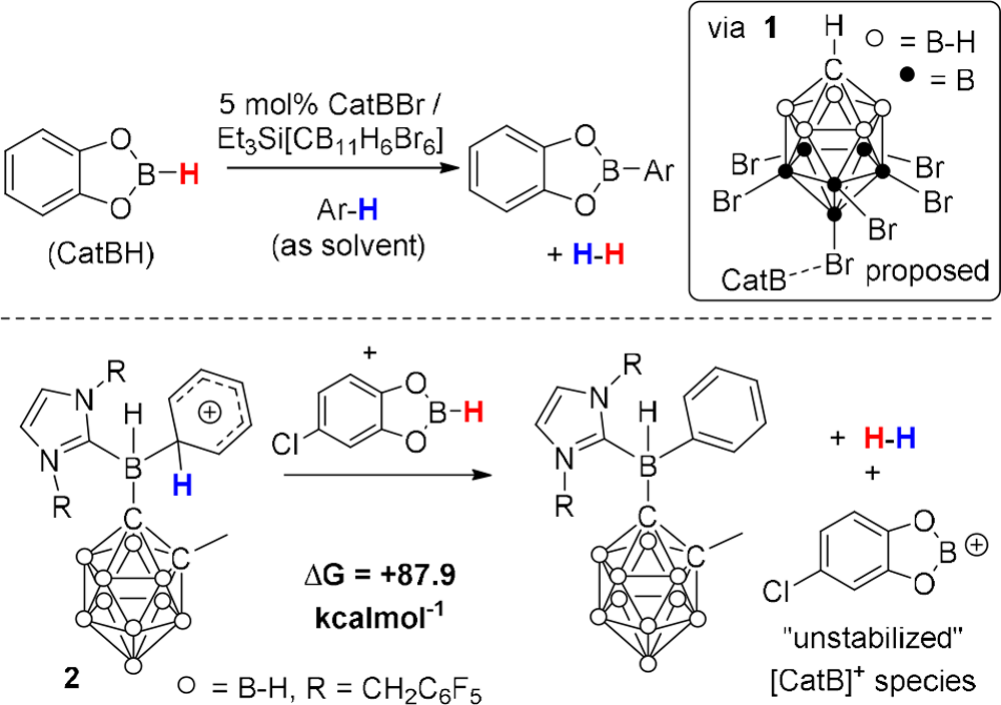
Top, Early Work on Catalytic Electrophilic Borylation. Bottom, Calculated Energy for the Dehydrocoupling Step Forming an Unstabilized [CatB]^+^

This approach generally uses substoichiometric additives to convert CatBH (or a derivative) into an electrophile that then effects arene C–H borylation. In our early work CatBH was combined with 5 mol % of CatBBr/[Et_3_Si][*closo*-CB_11_H_6_Br_6_].^13^ This led to a highly reactive, albeit ill-defined, boron electrophile that could even borylate deactivated aromatics with *N* values ≈ −7 (e.g., haloarenes). This study highlighted a number of important factors: (i) the need for a robust (toward electrophiles) hydroborane as pinacolborane decomposed under the reaction conditions; (ii) the necessity for the correct anion, with key considerations being low anion coordinating ability and high anion stability. In this work the highly reactive nature of the electrophile(s) necessitated use of the extremely robust [*closo*-CB_11_H_6_Br_6_]^−^ anion.^14^ The reactive boron species was termed [CatB(*closo*-CB_11_H_6_Br_6_)], **1** (Scheme 2, top inset), and was described as a synthetic equivalent of [CatB]^+^. Note, [CatB]^+^ is not feasible in the condensed phase without some further ligation of boron (e.g., by anion, solvent, and/or another base). The identity of this ligating species, termed L, is a key variable in approach (i) as it has a significant effect on multiple steps in the catalytic cycle.

Following the generation of the reactive electrophile **1**, arene C–H borylation proceeded generating H_2_ as the only byproduct.^13^ However, how borylation proceeded/H_2_ formed was not defined, with several possibilities discussed. One possible route to H_2_ formation is via a highly Brønsted acidic arenium salt (e.g., [C_6_H_6_E][CB_11_H_6_Br_6_], E = H or CatB, as an intermediate or a byproduct from S_E_Ar,), reacting with CatBH to reform the reactive electrophile (e.g., **1**). In this step the nature of the Brønsted acid and the hydroborane dramatically impacts the thermodynamics of the reaction to form H_2_ (termed dehydrocoupling). Recent calculations (Scheme 2, bottom) showed that the deprotonation of arenium cation **2** with a CatBH derivative to form an unstabilized [CatB]^+^ species was endergonic by +87.9 kcal mol^–1^.^15^ This is consistent with our original description of [CatB]^+^ as being unfeasible in the condensed phase, indicating a more complex dehydrocoupling process is occurring in this case.^13^

**Scheme 3:**
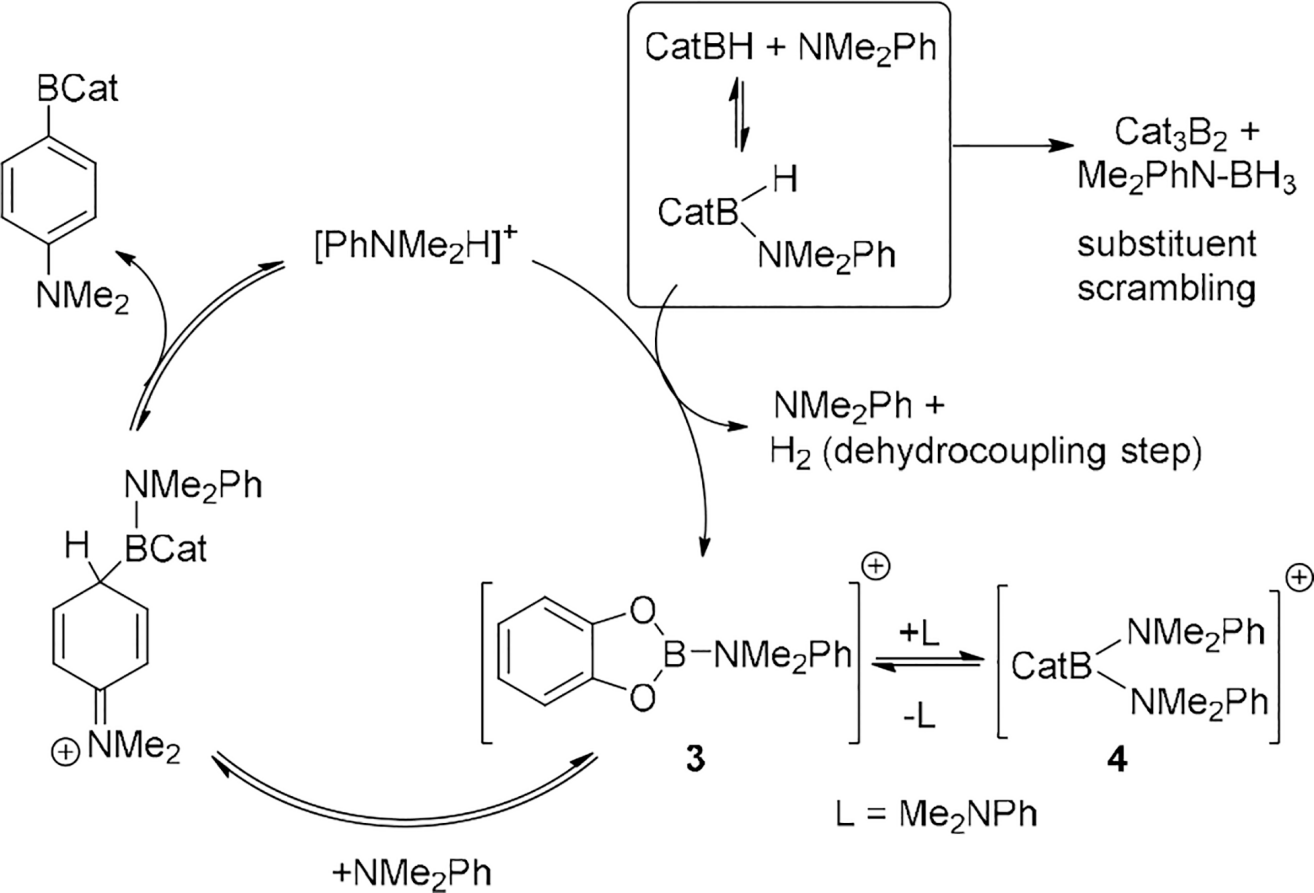
Potential Processes in [CatB–L]^+^ Mediated Catalytic Borylation This excludes anion involvement for simplicity; with, e.g. [HB(C_6_F_5_) _3_]^−^, more pathways are feasible.

Subsequent reports from multiple groups utilized CatBH/PinBH in the presence of exogenous L (which forms a dative bond with the hydroborane) to borylate arenes using a substoichiometric Lewis acid activator (e.g., B(C_6_F_5_)_3_ or a derivative thereof).^16^ These studies highlighted the dramatic effect L has on catalytic electrophilic borylation reactions. This is consistent with the multiple roles L can play in borylation cycles, for example: coordination of L to CatBH weakens the B–H bond facilitating hydride abstraction and formation of [CatB–L]^+^ cations, such as **3** (Scheme 3); (ii) the nature of L in the reactive [CatB–L]^+^ electrophile modulates the Lewis acidity at boron; (iii) L can also be the Brønsted base that deprotonates arenium cation intermediates; (iv) the conjugate acid, [LH]^+^, then is involved in the dehydrocoupling step forming H_2_ and regenerating the electrophile and L. A number of these processes are shown in Scheme 3 based on studies of Oestreich and co-workers.^16a^ It should be noted that L also can lead to off-cycle species, such as [CatB(L)_2_]^+^ boronium cations (e.g., **4**), and to substituent redistribution of CatBH to form Cat_3_B_2_ and L–BH_3_.^17^ Furthermore, in some systems competing reactivity (e.g., reduction of indoles to indolines) can occur to generate a “hidden” L *in situ* which can also play a crucial role.^16a,18^

Changing L can impact different steps in opposite ways; e.g., when L is a weak Lewis base it will bind weakly to CatBH and make hydride abstraction less favored enthalpically, but it will enhance the electrophilicity of [CatB–L]^+^. Furthermore, a weakly Brønsted basic L will lead to less exothermic deprotonation of arenium intermediates but generate a stronger conjugate Brønsted acid ([LH]^+^), thereby impacting the energetics of the dehydrocoupling step. The above only consider minima on the potential energy surface; clearly, altering L will impact the energy of the transition state involved in each step. Thus, it is challenging *a priori* to predict an optimal L for electrophilic borylation even when ignoring competing off-cycle reactivity, e.g., substituent scrambling and L binding to the activator. Nevertheless, given the range of bases studied to date, particularly with CatBH and using fluoroaryl-borane activators, there is limited potential to significantly improve reactivity in catalytic electrophilic borylation by utilizing other monofunctional bases.

**Scheme 4:**
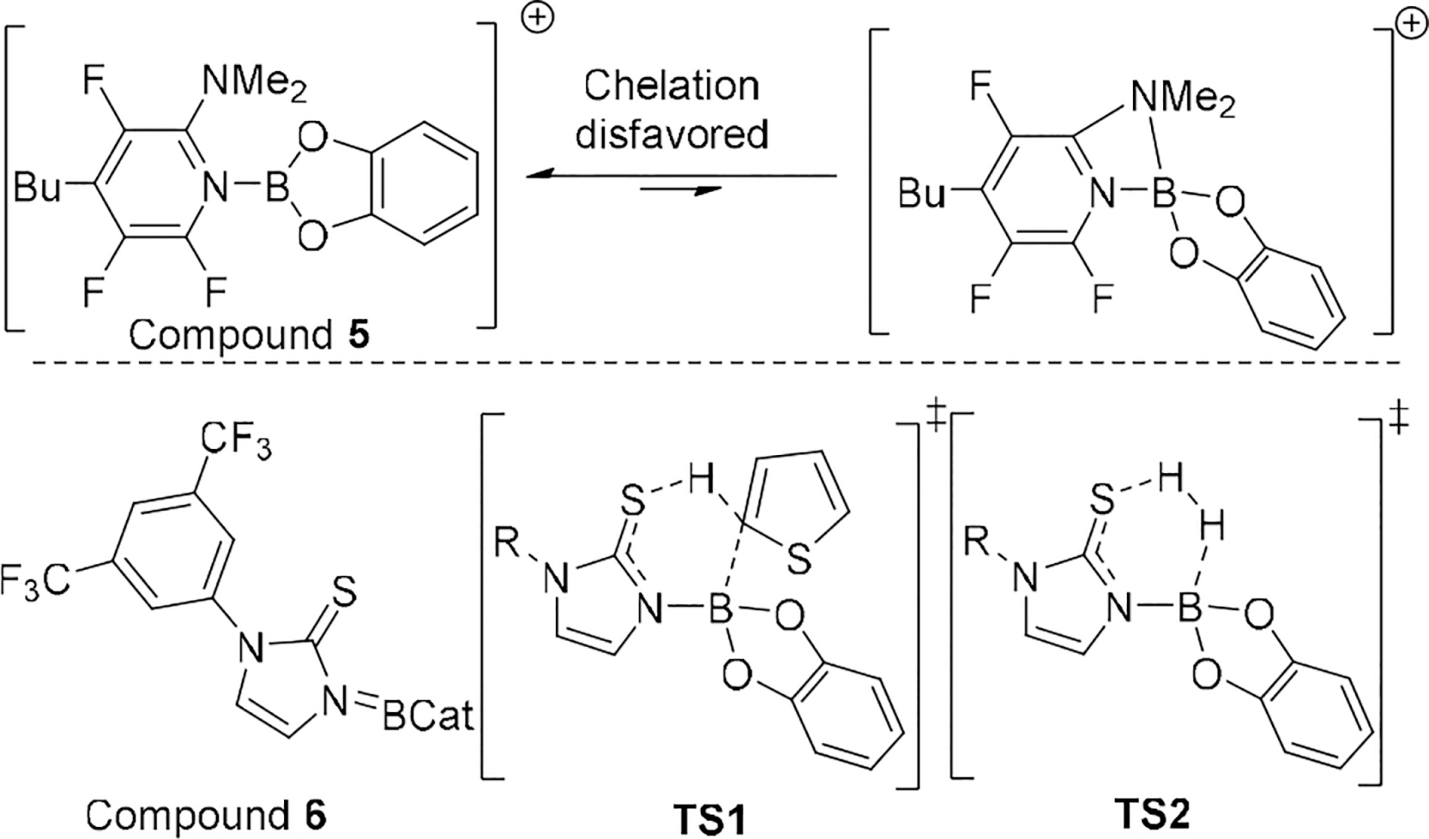
Top, Borenium and Boronium Forms of **5** and Bottom, Compound **6** and Key Transition States in the Catalytic Borylation Process Starting from Compound **6**

Moving beyond monofunctional bases in [CatB–L]^1+/0^ systems, bifunctional derivatives, e.g., 2-DMAP, also have been explored. While this was initially in stoichiometric C–H borylation,^19^ later work with compound **5** (Scheme 4)^20^ and more recently compounds such as **6** have shown that combining bifunctional bases with CatBH can expand the scope in catalytic electrophilic borylation to some degree.^21^ Chelation of bases such as 2-DMAP to {CatB} is disfavored as chelation would form a strained four-membered boracycle (Scheme 4, top).^19^ Instead the boron unit remains (or can readily become) three coordinate and sufficiently electrophilic to interact with arenes. The bifunctional nature of L is crucial as it introduces a basic site proximal to the electrophilic boron center (thus is a preorganized FLP). This facilitates C–H borylation via a six-membered transition state (**TS1**, Scheme 4) which is calculated to be the highest energy state in the process.^21^ The concerted nature of C–B formation/C–H deprotonation enabled by bifunctional bases has proved essential to expand the scope beyond that using monofunctional bases and CatBH. It is also notable that the optimal bifunctional base contains electron withdrawing groups. This presumably helps engender sufficient Lewis acidity at boron to lower **TS1** and sufficient Brønsted acidity in the conjugate acid post C–H borylation to enable the low barrier protonation of a CatBH(L) species via **TS2**. This again emphasizes that the selection of L requires a balancing act to prevent any one transition state becoming too high in energy. To date the systems in Scheme 4 are effective for catalyzing the borylation of heteroarenes with *N* values ≥ −1.5; however, benzothiophene (*N* = −2.6) did not undergo borylation. With fewer reports using bifunctional bases, it is possible that improved bifunctional base/hydroborane combinations exist that will increase the scope further. The studies using **6** also highlight the complementary nature of catalytic electrophilic borylation, with groups often challenging in precious metal catalyzed borylation (e.g., alkenes, R_2_S) tolerated. Furthermore, hindered sites can be borylated with **6** (e.g., C2 in 3-R-thiophenes),^21^ whereas iridium catalyzed borylation proceeds under steric control.

**Scheme 5:**
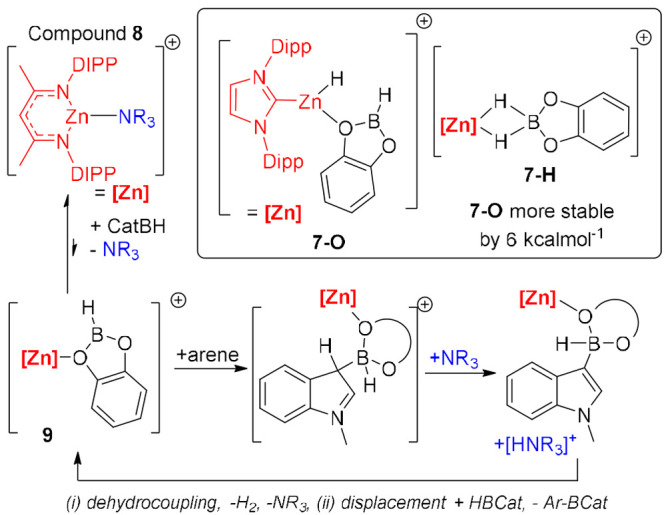
Zinc Electrophile Binding to HBCat (inset O vs B–H) and Borylation Mediated by This Electrophile Type

The systems discussed above all form (or are proposed to form) [CatB–L]^1+/0^ species as the key electrophile effecting C–H functionalization. An alternative route to generate reactive electrophiles from CatBH is by binding a Lewis acid to an O or the B–H. As the Lewis acidity in dioxaborolanes is significantly affected by the π donor ability of the oxo groups,^22^ attaching a Lewis acid at O will modify the Lewis acidity at boron. Our recent work utilized this approach in catalytic arene borylation with low coordinate zinc cations calculated to prefer binding to O over the B–H unit (Scheme 5 inset, **7-O** vs **7-H**).^23^ Using 10 mol % of compound **8** (Scheme 5) as a precatalyst enabled catalytic borylation and an increase in the arene scope relative to [CatB–L]^1+/0^ mediated catalytic borylation, with benzothiophene now amenable to C–H borylation.^24^ Computational analysis revealed a mechanism initiated by an endergonic displacement of amine NR_3_ from zinc by CatBH to form **9**. **9** represents a borenium equivalent and is proposed to form an FLP with the released amine. The borylation then proceeds via arenium cation formation, with this cation deprotonated by the amine, with the formed ammonium cation then undergoing dehydrocoupling with the borohydride. Finally, displacement of CatB–Ar by CatBH closes the cycle (Scheme 5, bottom). This reaction mechanism has an overall free energy span of 22.4 kcal mol^–1^ for *N*-Me-indole, consistent with the room temperature reactivity observed. The scope of this process was found to be affected by the base used and also by the nature of the zinc cation, indicating that varying the activating metal electrophile that binds to CatBH can have a dramatic effect on reactivity.^23,24^

**Scheme 6:**
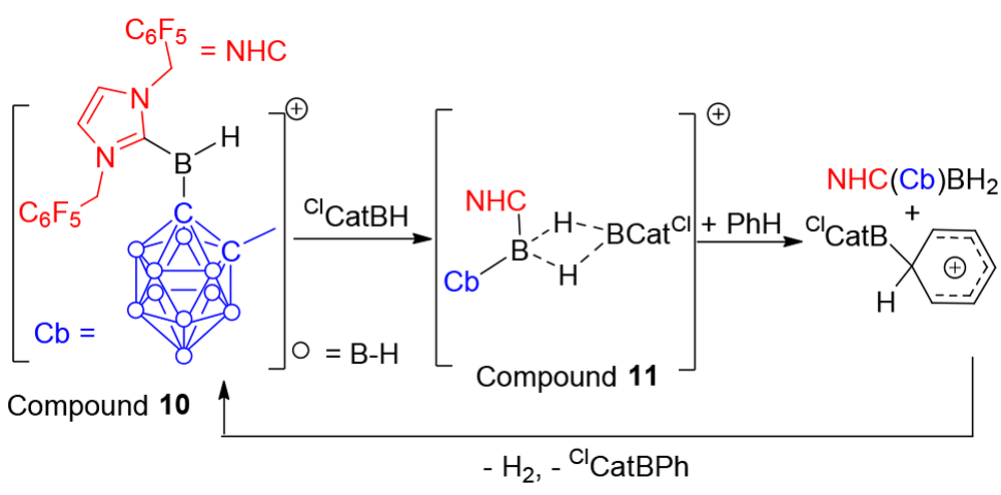
Borylation Catalyzed by Borenium Cation **10**

In recent work, Wang and co-workers reported the use of the borenium cation **10** (Scheme 6) as the electrophilic activator.^15^**10** activated 4-chloro-CatBH^25^ to form an electrophile that was highly effective in arene borylation. Compound **10** (5 mol %) was even sufficient to borylate benzene (*N* value = −6.3) in high yield with ^Cl^CatBH. A highly electrophilic borenium cation is essential with B(C_6_F_5_)_3_, and a less Lewis acidic borenium cation not effective. **10** was proposed to interact with the B–H of ^Cl^CatBH to generate electrophile **11** (Scheme 6). **11** then reacts with arenes by cleavage of both of the 3-center, 2-electron bonds and formation of a ^Cl^CatB-substituted arenium cation. This is deprotonated by the NHC-borane, evolving H_2_ and reforming **10**; notably, this combines deprotonation and dehydrocoupling into one step. This last step is calculated to be rate limiting, consistent with the measured KIE of 2.8. In addition to borylating weakly nucleophilic arenes, this work also borylated phenols selectively at the para position, with no meta borylation observed (in contrast to iridium catalysis which gives a mix of meta and para borylation).^26^ Furthermore, sterically hindered arenes, e.g., mesitylene, also underwent borylation using **10** in good yield; note, these hindered substrates are challenging for iridium catalysts. This work combined with the zinc-catalyzed systems indicates that electrophile binding to CatBH (and derivatives) to generate borocation equivalents is an underexplored area that may lead to further advances in catalytic borylation.

**Scheme 7:**
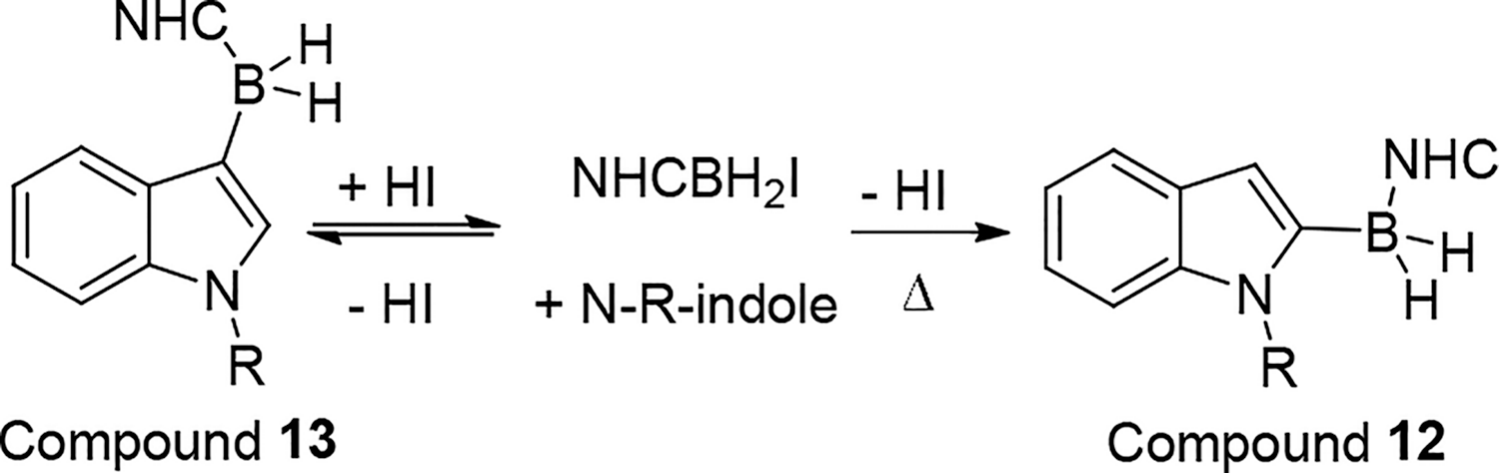
Formation of Borylated Products **12** and **13**

Alongside increasing the scope, switching the regioselectivity in catalytic electrophilic borylation is also of interest. To date, the vast majority of reports generate the products expected from S_E_Ar. In contrast, the borylation of ethylbenzene with ^Cl^CatBH/**10** gave ca. 1:1 mixtures of C4 and C3 borylated products.^15^ This is potentially due to isomerization at the borylated arenium cation stage prior to deprotonation as observed in stoichiometric electrophilic borylation.^27^ Controlling the rate of deprotonation relative to that of isomerization therefore offers a route to obtain distinct borylation products. Analogously, if deprotonation of the “kinetic borylated-arenium cation” is reversible, then the thermodynamic borylated product could be accessed selectively. The latter was observed in our work on borylations using NHC-BH_3_/10 mol % I_2_.^28^ This forms NHC–BH_2_I that reacts with activated arenes by S_E_Ar, with reaction of the HI byproduct with NHC–BH_3_ regenerating NHC–BH_2_I. With *N*-Me-indole, the only product observed was the C2 borylated product **12** (Scheme 7), with C3 borylation expected from the S_E_Ar of indoles. The expected S_E_Ar product, **13**, was formed selectively in the presence of a stronger (than iodide) Brønsted base. However, in the absence of a base **13** was formed reversibly, ultimately leading to formation of **12** as the only borylated product, consistent with **12** being calculated to be the thermodynamic product. Hatakeyama and co-workers also noted that, in borylations using BI_3_ in the absence of a base, reversible borylation occurs, presumably enabled by the HI byproduct from S_E_Ar.^29^

These studies with NHC boranes also emphasize that boranes other than dioxaborolanes can be useful in catalyzed electrophilic borylations. This is an underexplored area and one that may lead to distinct reactivity/products. This is exemplified by the use of BF_3_/hindered amine FLPs to borylate activated arenes. This enabled formation of trifluoroorganoborates by electrophilic borylation. Notably, the addition of 5 mol % tetramethylthiourea (TMTU) catalyzed this borylation process, possibly by providing a lower barrier deprotonation pathway or by enabling formation of different reactive electrophiles, e.g., [(TMTU)BF_2_]^+^ cations.^30^

## Approach (ii) Arene C–H Functionalization Requiring a σ-Bond Metathesis Step

**Scheme 8:**
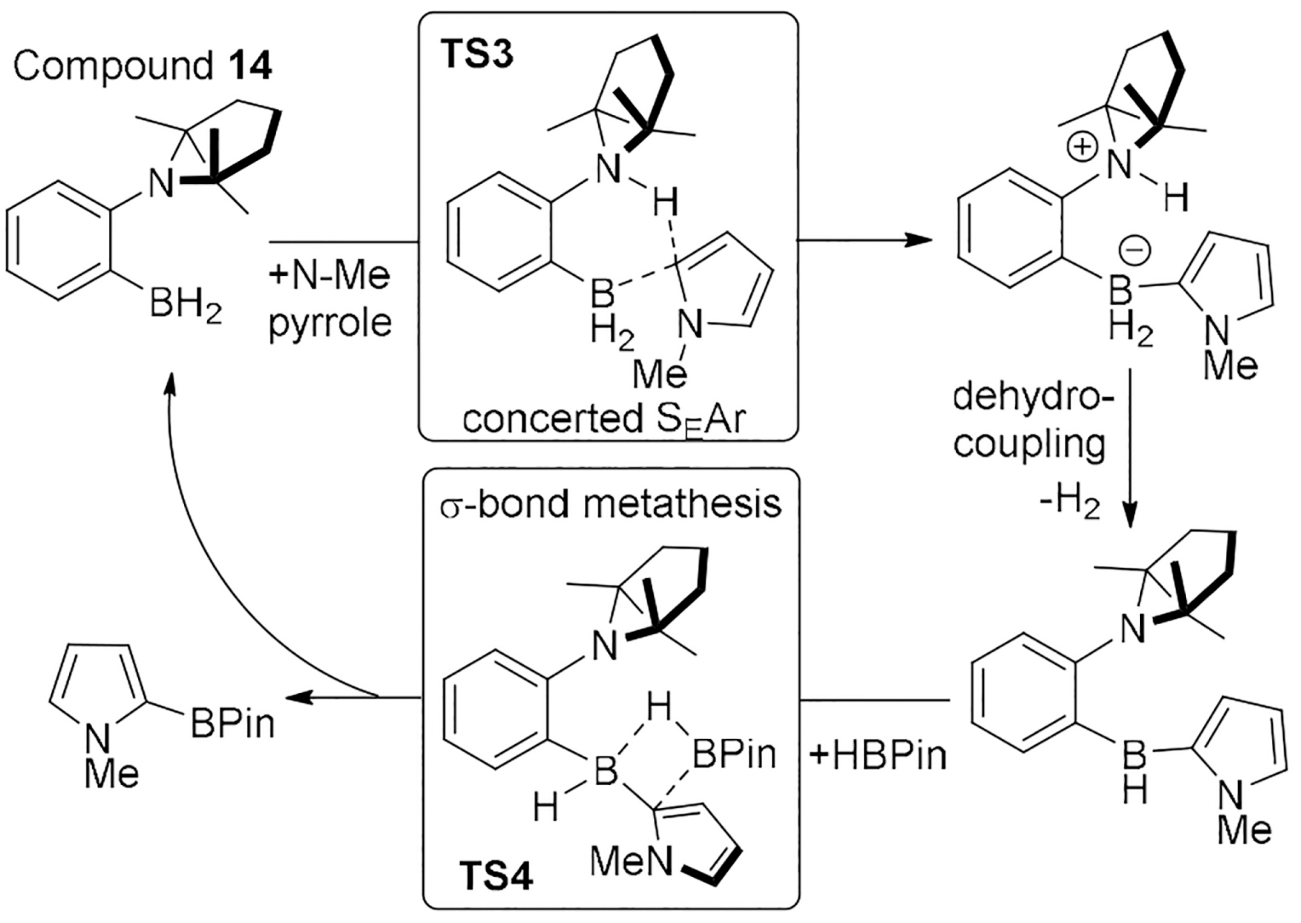
FLP C–H Functionalization, Dehydrocoupling, and σ-Bond Metathesis Combined into a Catalytic C–H Borylation

Building on breakthroughs in FLP chemistry,^31^ in 2015 Fontaine and co-workers reported an FLP that catalyzed arene C–H borylation with PinBH.^32^ Vital to this process was linking a C–H functionalization step with a σ-bond metathesis step (Scheme 8). C–H functionalization using **14** occurs under milder conditions than that using hydroboranes that do not contain a pendant base.^33^ Consistent with this, a single borylation–deprotonation transition state was calculated using **14** (**TS3**), which can be viewed as a concerted S_E_Ar type process.^9^ The concerted transition states accessible in systems containing pendant bases enables what are relatively weak (compared to boreniums) boron electrophiles to effect C–H functionalization. Presumably, this is due to the preorganized nature of unimolecular FLPs circumventing arenium intermediates and a separate deprotonation step with an exogenous base (which has a significant entropic penalty). It should be noted that an initial endergonic step to form the active FLP **14** is required, which can be termed a FLP preparation step. This step is also present in derivatives of **14** and when using **6** and **9**. It is associated with dimer dissociation/cleavage of a dative bond to access the FLP. This step has the effect of making the subsequent energy profile more endergonic, with the preparation energy required generally greater the lower the steric bulk is in the FLP. Therefore, developing systems where the active FLP is the resting state would be attractive for increasing the substrate scope (as less nucleophilic arenes have higher C–H functionalization barriers which combined with the FLP preparation energy can preclude reactivity).

**Scheme 9:**
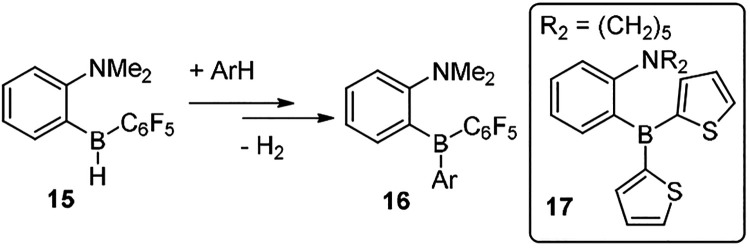
C–H Functionalization with the More Lewis Acidic FLP **15** Compounds **16** and inset **17** have not been reported to undergo σ-bond metathesis with hydroboranes to our knowledge.

Structure activity studies from the groups of Repo^20^ and Fontaine^34^ revealed that tuning the boron substituents and/or reducing the steric bulk around N can reduce the barrier to C–H functionalization. A significant impact on this step was observed by altering the substituents at boron to generate more Lewis acidic FLPs, with **15** (Scheme 9) able to effect C–H functionalization of benzene in contrast to the BH_2_ analogues.^20^ However, while C–H activation and H_2_ evolution are achieved starting from **15** to form compounds of general formula **16**, the σ-bond metathesis step between **16** and a hydroborane has not been reported to date to our knowledge. The σ-bond metathesis step is unique to approach (ii) and is a key obstacle that has prevented significant substrate expansion via this approach. This is also highlighted by the nucleophilic heteroarene thiophene proving challenging to borylate catalytically using **14** and PinBH.^12a^ In this case double C–H functionalization occurs, e.g., to form **17**, but **17** does not undergo σ-bond metathesis with PinBH. To date successful σ-bond metathesis, and thus turnover via approach (ii), has been limited to FLP catalysts containing BH_2_ units (e.g., **14**). Finding alternative hydroboranes (to PinBH or CatBH) that undergo selective σ-bond metathesis with compounds such as **16** could significantly expand the substrate scope via this approach, as would identifying distinct BH_2_-containing FLPs that are more Lewis acidic than **14** (to enable C–H functionalization of less nucleophilic arenes) but still undergo metathesis with PinBH.

**Scheme 10:**
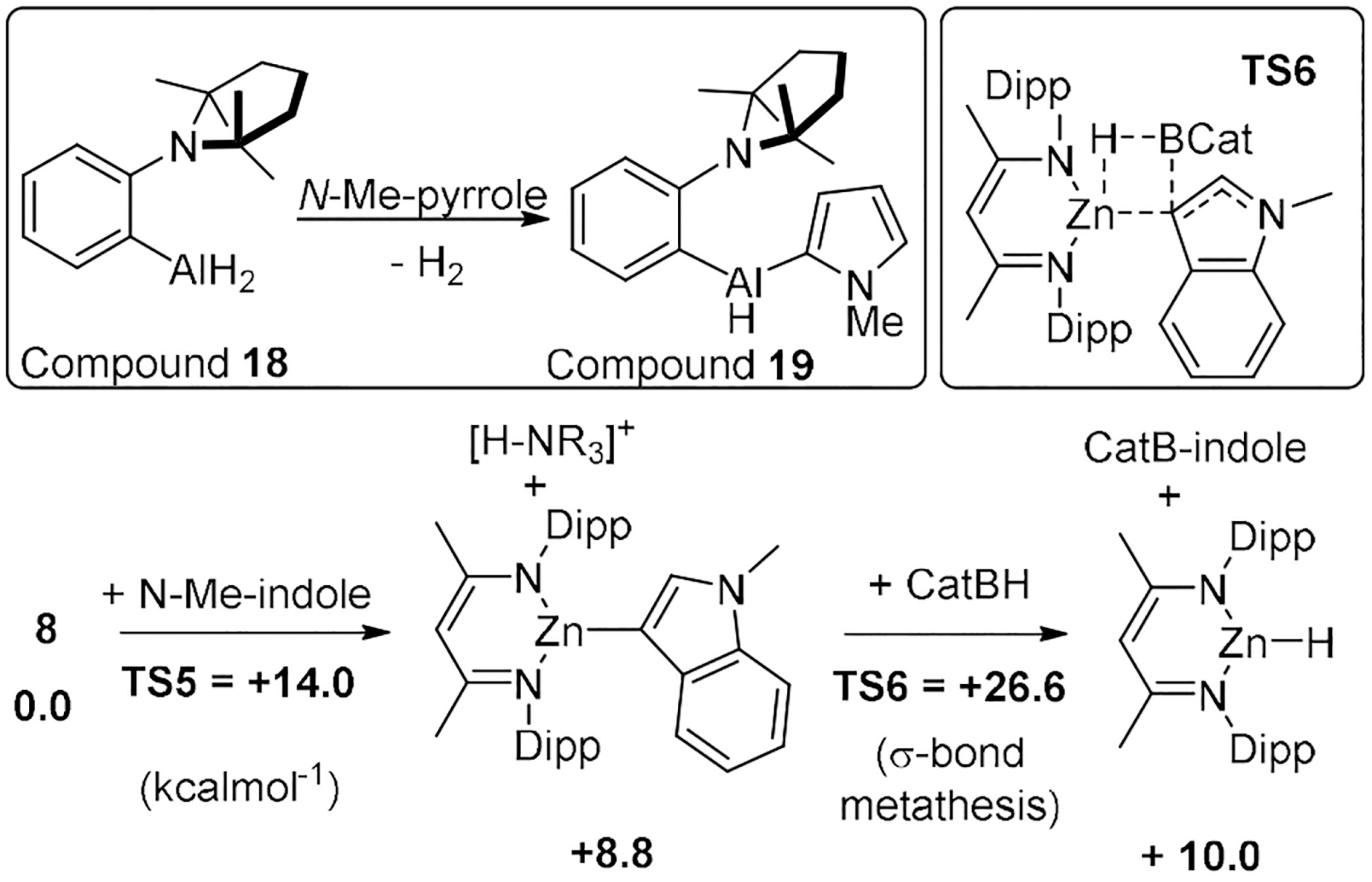
Inset Top, FLP Mediated Alumination. Inset, Top Right, **TS6**. Bottom, Calculated Mechanism Starting from **8** That Proceeds via Zincation and Then σ-Bond Metathesis

Analogues of **14** based on aluminum, e.g., **18** (Scheme 10 inset top), also have been shown to effect heteroarene C–H functionalization and undergo dehydrocoupling.^35^ However, the metathesis step of species such as **19** with PinBH or CatBH has not been reported to date to our knowledge, suggesting it either does not proceed or leads to complex mixtures. It should be noted that a system closely related to **18** (with a NMe_2_ unit) does catalyze terminal alkyne C–H borylation with PinBH.^36^ However, many main group compounds have been reported that catalyze alkyne C–H borylation, and this conversion is in general less challenging than arene C–H borylation. Finally, in this section, it is noteworthy that a zinc catalyzed C–H functionalization/metathesis mechanism also was explored computationally starting from compound **8**.^24^ This had an overall free energy span for the borylation of *N*-Me-indole of 26.6 kcal mol^–1^ (Scheme 10 bottom), in comparison to 22.4 kcalmol^–1^ for that via approach (i) (shown in Scheme 5). Note, the cycle in Scheme 10 would be closed by the protonated amine reacting with NacNacZnH (a process that was calculated to have a low barrier and be highly exergonic). Notably, the major contributor to the overall barrier to catalytic C–H borylation via this approach is from the σ-bond metathesis step (**TS6**, Scheme 10, inset top right) and not the C–H metalation (**TS5**) step. Thus, exploring other hydroboranes (beyond the usual suspects of PinBH and CatBH) may facilitate this step and enable catalytic borylation via this approach.

## Operationally Simple Methodologies

**Scheme 11:**
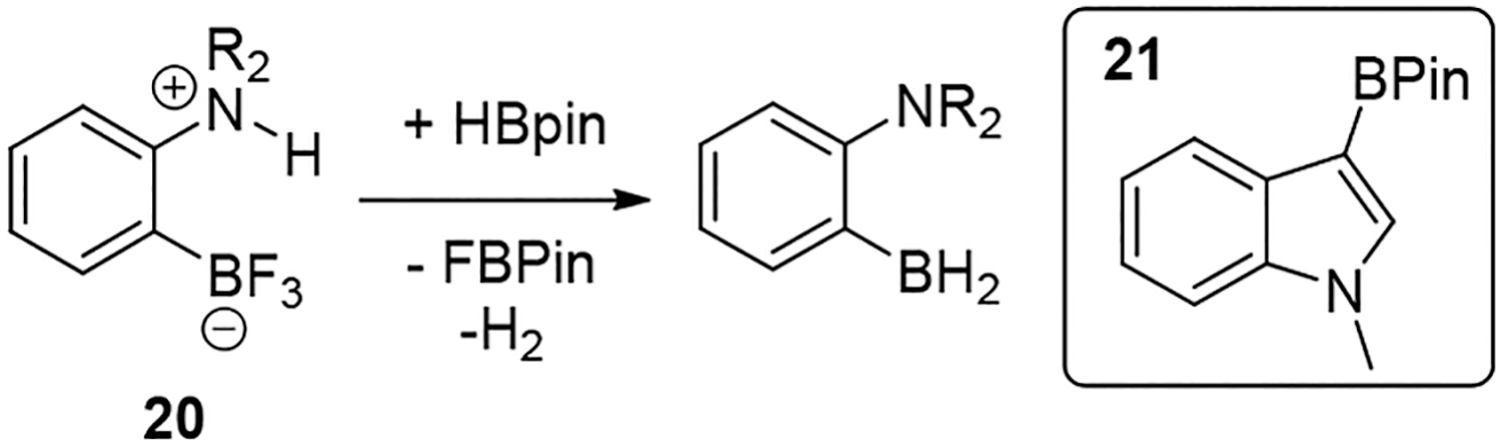
Bench Stable Zwitterion Precatalyst **20**. Inset, Compound **21** Produced on a Kilo Scale Using Precatalyst **20**

For a process to be widely used, it not only requires broad applicability, but it also needs to be operationally simple. This requires (pre)catalysts and reagents that are easy to handle (ideally bench stable). This actually excludes CatBH due to its sensitivity (to moisture) and its tendency to undergo substituent scrambling (e.g., to B_2_Cat_3_); thus, PinBH is clearly preferable. In this area, Fontaine and co-workers have progressed from their initial sensitive (pre)catalysts to bench stable zwitterionic precursors of general formula **20** (Scheme 11).^37^ The combination of **20** with bench stable PinBH led to formation of the active FLP *in situ* with PinB-F and H_2_ as the byproducts. This approach also was shown to be viable on a large scale. For example, precatalyst **20** (where NR_2_ = piperidine) could be synthesized on a >100 g scale and was effective in the borylation of a range of activated heteroarenes on scales up to 1 kg (for **21**).

Other groups also have developed operationally simple main group catalyzed borylation processes. For example, Zhang and co-workers avoided the complex zinc cations shown in Scheme 5, instead using Zn(OTf)_2_ to achieve the C3 borylation of *N*-Me-indoles using PinBH.^38^ Furthermore, the same group achieved the C2 borylation of *N*-alkyl-indoles using PinBH with substoichiometric PhCO_2_H as an activator, with the intermediacy of L–BH_3_ species derived from PinBH by substituent scrambling proposed.^39^

## Conclusions and Future Outlook

Given the short time span since the 2010 report using **1** in catalytic electrophilic borylation of arenes, notable advances have been made. However, for the wide uptake of main group catalyzed arene borylation an operationally simple system with broad scope is required. To date this has not been realized, although recent breakthroughs have brought it closer. These breakthroughs also have highlighted a number of pathways worthy of further exploration which include for approach (i): (a) utilizing bifunctional bases; (b) accessing boron electrophiles by binding the activator to an O or the B–H to form borenium equivalents; (c) minimizing the preparation energy required to access active FLPs; (d) controlling the relative rates of deprotonation versus isomerization to access alternative borylation regioisomers. For approach (ii) many of the considerations are the same as in (i), but this approach would also benefit from more diverse FLPs (e.g., systems not based on boron Lewis acids and not using a 1-amino-2-BR_2_-phenyl structure) being explored. This may help solve the current challenge associated with the σ-bond metathesis step that currently limits the substrate scope in catalytic electrophilic borylation via approach (ii). Breakthroughs have also been reported using boron electrophiles outside of the usual dioxaborolane based systems. While this is an underexplored area that can enable novel chemistry, it does introduce an additional consideration—the primary products from C–H borylation need to be synthetically useful in their own right, or they must be able to be converted readily into useful organoboranes, e.g., pinacol boronate esters.

**Scheme 12:**
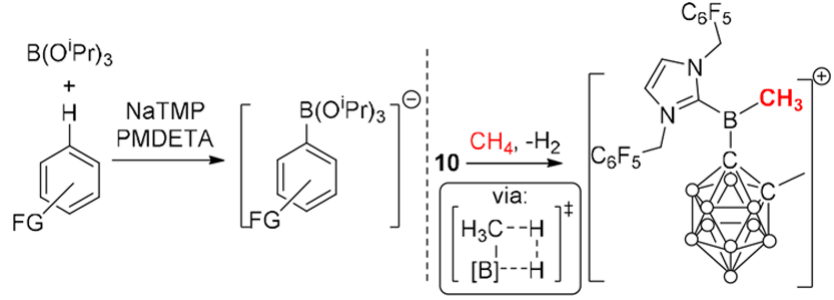
: Left, Strong Base Mediated Metalation/Borylation. Right, C–H Borylation of CH_4_ Using a Borenium Cation

Moving forward, the fact that the catalytic approaches discussed above all rely on electrophile based C–H functionalization steps is a limitation. It would be extremely interesting if fundamentally different main group catalyzed arene borylation routes could be developed. For example, is a strong Brønsted base mediated catalytic borylation process feasible? Conceptually this would proceed via a strongly basic main group compound metallating (deprotonating) an arene, and this would be followed by reaction of the Aryl-M product with an appropriate borane to form the arylborane, ideally with concomitant regeneration of the strong Brønsted base. A step toward this has been reported recently by Hevia and co-workers (left, Scheme 12) who used NaTMP and B(O^i^Pr)_3_ (TMP = 2,2,6,6-tetramethylpiperidine) in the presence of PMDETA (PMDETA= *N,N,N*′*,N*″*,N*″-pentamethyldiethylenetriamine) to effect C–H borylation.^40^ The borylation proceeds via a C–H metalation step to form an aryl–sodium complex that then arylates B(O^i^Pr)_3_, with the latter step key to drive the C–H metalation to completion. This work is an extension beyond the classical stepwise ArylC–H metalation/B(OR)_3_ trapping—as NaTMP and B(O^i^Pr)_3_ are mutually compatible, i.e., they do not react irreversibly with each other. If a related system could be made to turnover, then it would be complementary to catalytic electrophilic borylation methods. The complementarity is indicated by the stoichiometric NaTMP/B(O^i^Pr)_3_ system being able to borylate the highly deactivated (in term of nucleophilicity) arene PhCF_3_. However, there are still significant challenges to overcome to combine metalation with a subsequent step (e.g., metathesis with a borane)^41^ that would regenerate the strong base and close the cycle.

Finally, Vedejs’ seminal work on intramolecular borylation offers a different mechanistic pathway to achieve C–H borylation.^42^ This proceeds via a C–H/B–H σ-bond metathesis type transition state (inset, Scheme 12). This approach, while demonstrated in intramolecular catalytic borylation chemistry,^43^ is underexplored in intermolecular borylation, but it has significant potential. The potential is best demonstrated by the recent report from Wang and co-workers on the borylation of methane with a borenium cation (right, Scheme 12).^44^ While this was not catalytic (in the electrophilic activator), it does indicate that with the right system main group catalyzed electrophilic alkane borylation is possible, which would be a highly notable breakthrough.
